# Supplementary material for: Monitoring transmission intensity of trachoma with serology: a multi-country study
Source: medRxiv. 2023 Feb 16:2023.02.13.23285881. Preprint. [Version 1] doi: 10.1101/2023.02.13.23285881 (PMC9949201; doi:10.1101/2023.02.13.23285881)
Supplement: Supplementary file 1 [file media-1.pdf]

Monitoring transmission intensity of trachoma with serology: a multi-country study

Tedijanto et al.

Supplementary Information Materials

Supplementary Fig. 1. Distribution of cluster sample sizes by study population.

Supplementary Fig. 2. Age distribution of children sampled by study population.

Supplementary Fig. 3. Correlation between seroconversion rates without seroreversion and seroconversion estimated using a semiparametric spline.

Supplementary Fig. 4. Relationship between seroconversion rates and PCR prevalence (a) in the presence and absence of recent MDA and (b) among 1–9-year-olds compared to 1–5-year-olds.

Supplementary Fig. 5. Identification of clusters with *C. trachomatis* infection using the seroconversion rate.

Supplementary Fig. 6. Identification of clusters with *C. trachomatis* infection using seroprevalence among 1-5 year olds.

Supplementary Fig. 7. Robustness of seroprevalence and seroconversion rate to changes in seropositivity threshold.

Supplementary Fig. 8. AIC values for simple catalytic model, reversible catalytic models with a range of assumed seroreversion rates, and semiparametric spline (SS) model for each study-year.

Supplementary Table 1. Overview of included studies.

Supplementary Table 2. Sensitivity and specificity to identify clusters with *C. trachomatis* infection by seroprevalence threshold among 1-9 year olds.

Supplementary Table 3. Sensitivity and specificity to identify clusters with *C. trachomatis* infection by seroconversion rate threshold among 1-9 year olds.

Supplementary Table 4. Sensitivity and specificity to identify clusters with *C. trachomatis* infection by seroprevalence threshold among 1-5 year olds.

Supplementary Table 5. Sensitivity and specificity to identify clusters with *C. trachomatis* infection by seroconversion rate threshold among 1-5 year olds.

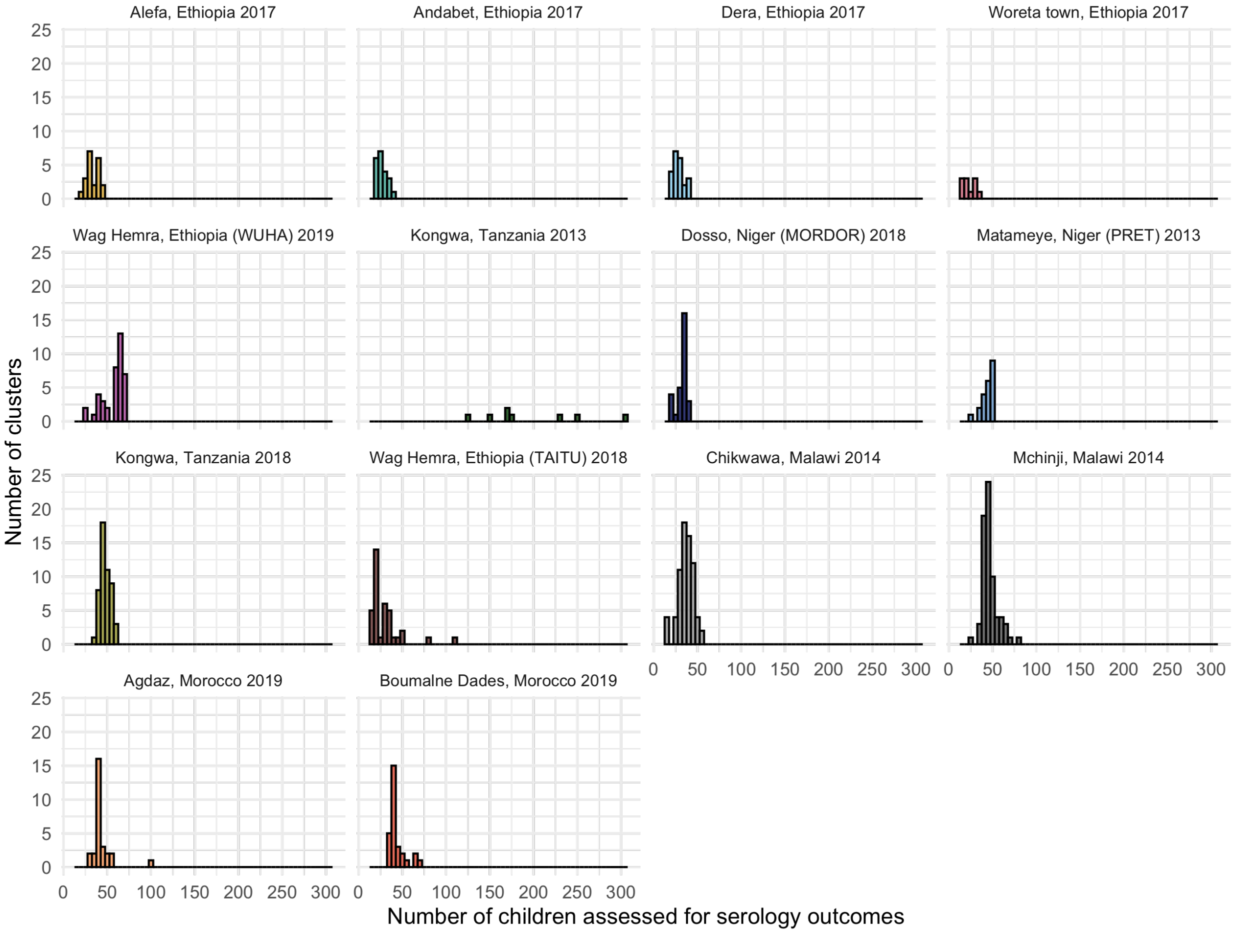

Supplementary Fig. 1. Distribution of cluster sample sizes by study population.

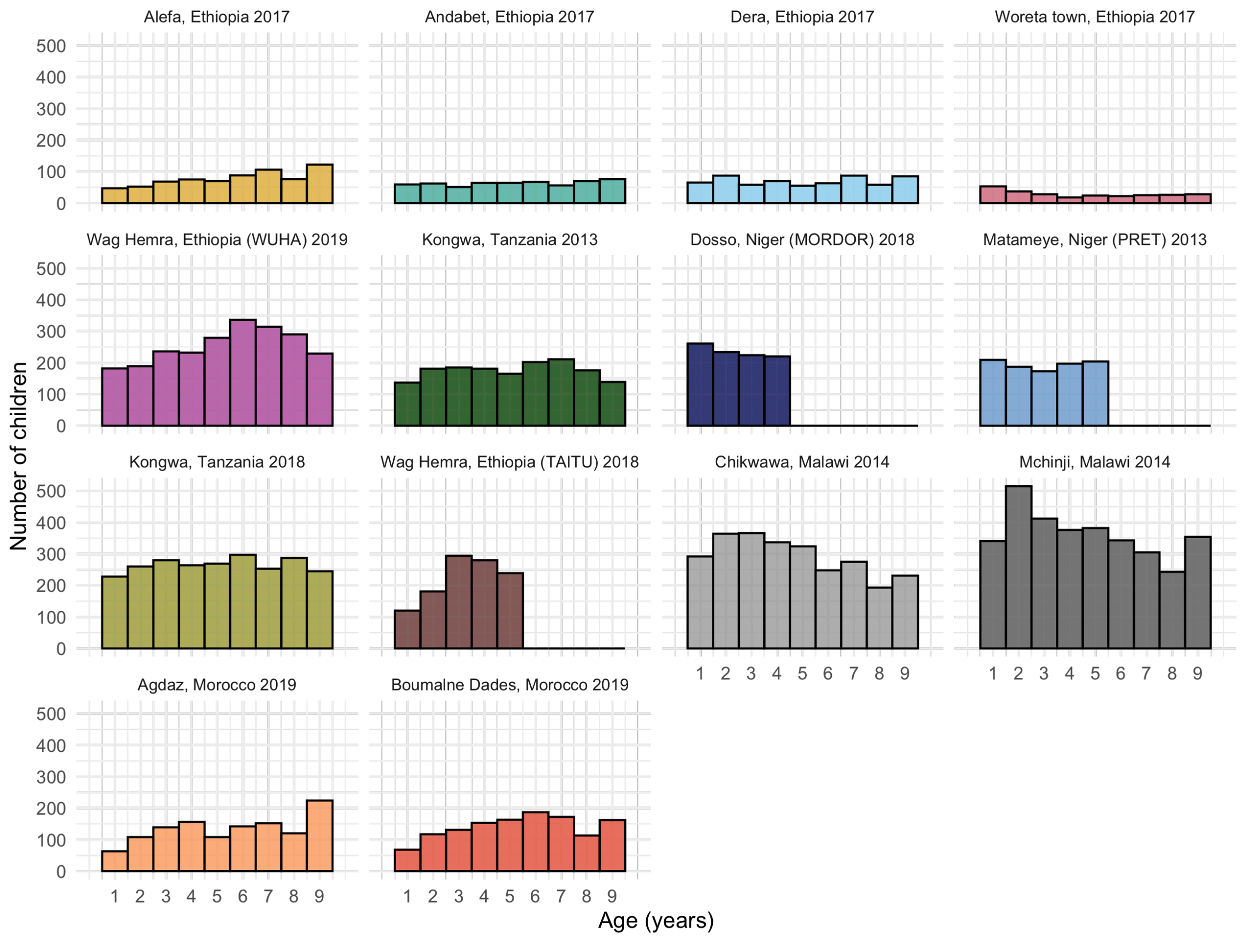

Supplementary Fig. 2. Age distribution of children sampled by study population.

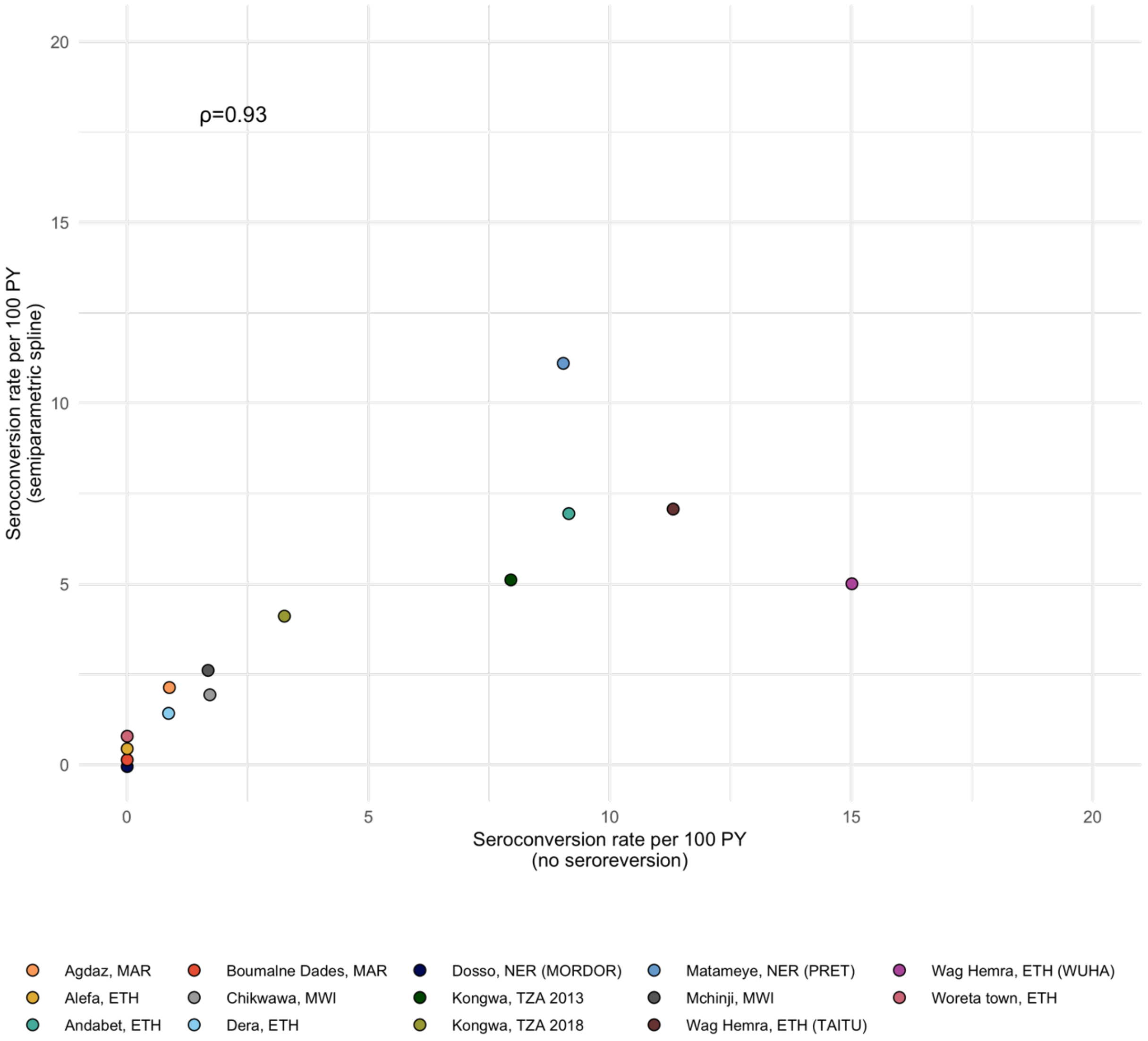

Supplementary Fig. 3. Correlation between seroconversion rates without seroreversion and seroconversion estimated using a semiparametric spline. Spearman rank correlation is shown at top left. Due to sample size considerations, seroconversion using semiparametric spline was estimated at the population level only.

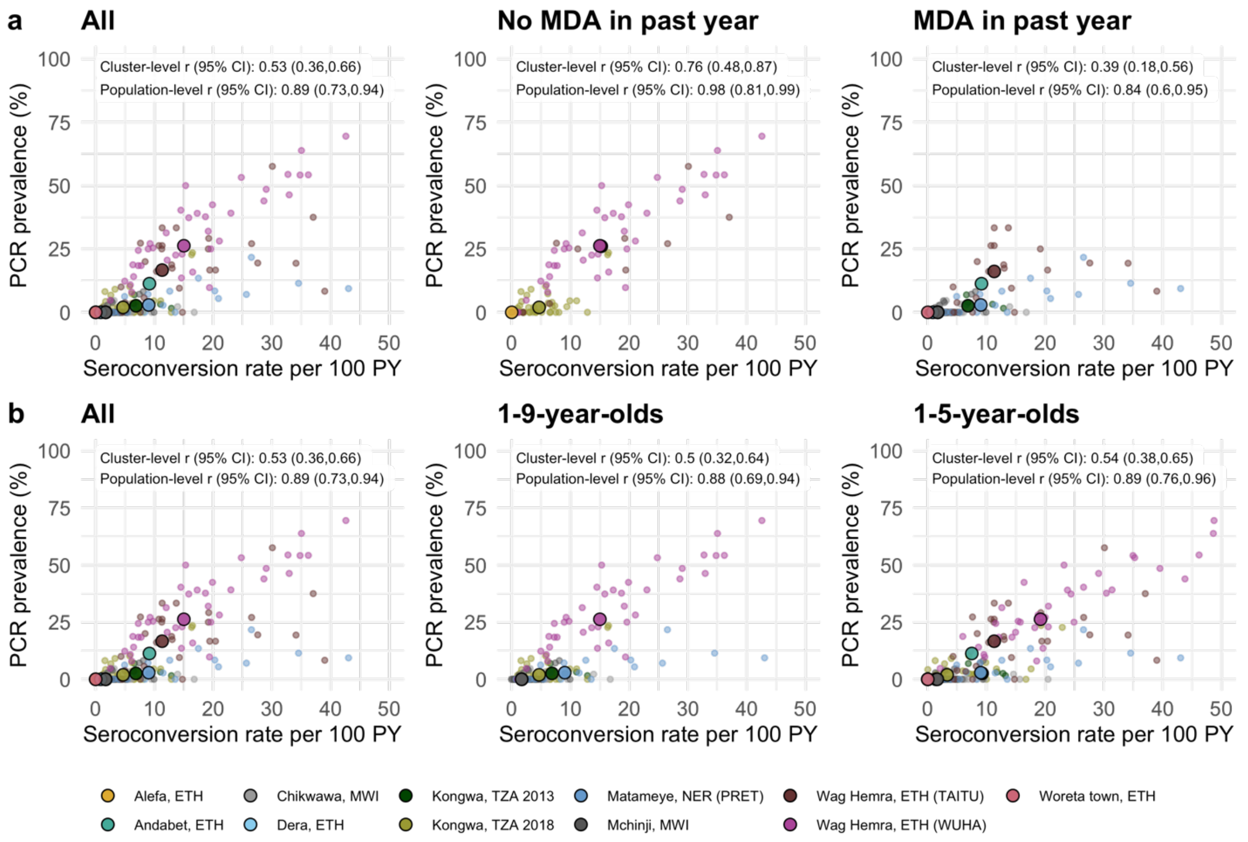

Supplementary Fig. 4. Relationship between seroconversion rates and PCR prevalence (a) in the presence and absence of recent MDA and (b) among 1–9-year-olds compared to 1–5-year-olds. Medians across clusters for each study population are represented by larger points with black outline. Each plot includes the identity line (dotted) and Pearson correlations at the cluster- and population-levels. 95% confidence intervals (CIs) are based on 1000 bootstrapped samples, holding populations fixed and resampling clusters with replacement. Population-level values are included for Andabet, Dera, Woreta town, and Alefa, Ethiopia, but cluster-level PCR prevalence was not available for these populations. For some populations including 1–9-year-olds, measurements were already summarized at the population-level and prevalence could not be separately estimated among 1–5-year-olds.

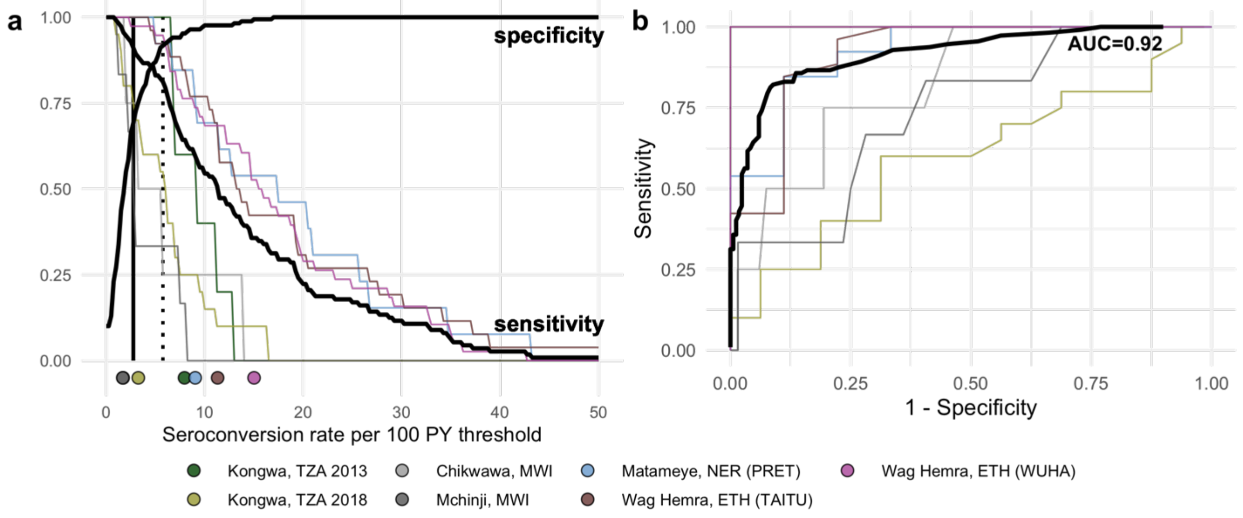

Abbreviations: ETH = Ethiopia; NER = Niger; TZA = Tanzania; MWI = Malawi

Supplementary Fig. 5. Identification of clusters with *C. trachomatis* infection using the seroconversion rate.
(**a**) Sensitivity (red) and specificity (blue) for identification of clusters with any *Ct* infections based on different seroconversion rate thresholds among children 1-9 years old. Vertical lines delineate sensitivity at 90% (solid line, 2.75 seroconversions per 100 PY) and 80% (dotted line, 5.75 seroconversion per 100 PY). The thick lines represent overall sensitivity and specificity (n = 281 clusters; 99 clusters with PCR>0%), while thin lines show sensitivity for each study population. Points at bottom of plot mark seroconversion rate estimates for each study population. (**b**) Receiver operating characteristic (ROC) curves for all clusters (thick black line) and for each population (thin colored lines) with overall area-under-the-curve (AUC).

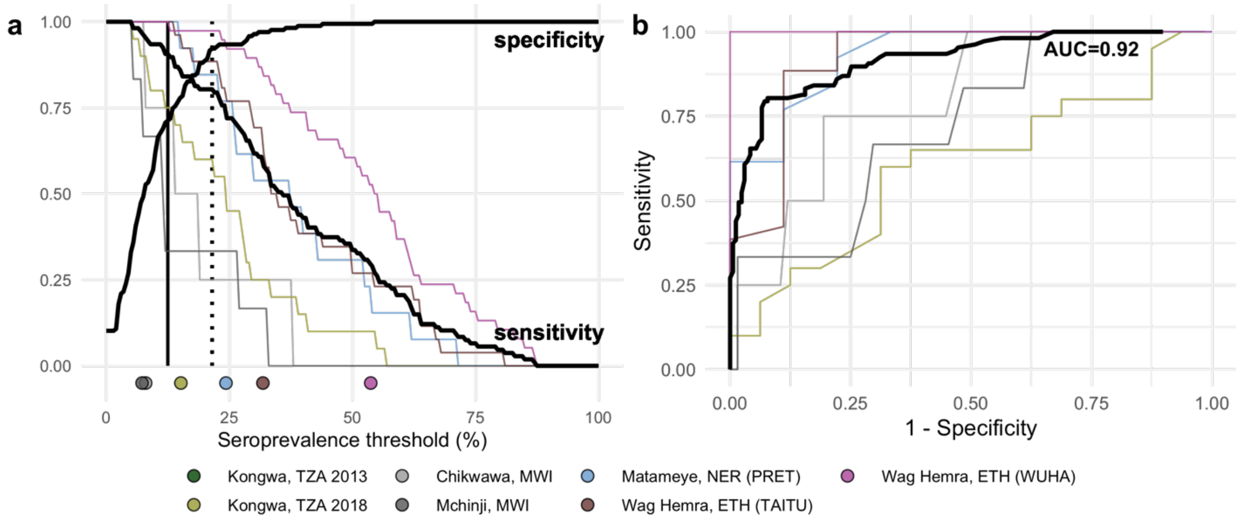

Abbreviations: ETH = Ethiopia; NER = Niger; TZA = Tanzania; MWI = Malawi

Supplementary Fig. 6. Identification of clusters with *C. trachomatis* infection using seroprevalence among children 1–5 years old.

(**a**) Sensitivity (red) and specificity (blue) for identification of clusters with any *Ct* infections based on different seroprevalence thresholds among children 1-5 years old. Vertical lines delineate sensitivity at 90% (solid line, 12.5% seroprevalence) and 80% (dotted line, 21.5% seroprevalence). The thick lines represent overall sensitivity and specificity (n = 274 clusters; 94 clusters with PCR>0%), while thin lines show sensitivity for each study population. Points at bottom of plot mark seroprevalence estimates for each study population. (**b**) Receiver operating characteristic (ROC) curves for all clusters (thick black line) and for each population (thin colored lines) with overall area-under-the-curve (AUC).

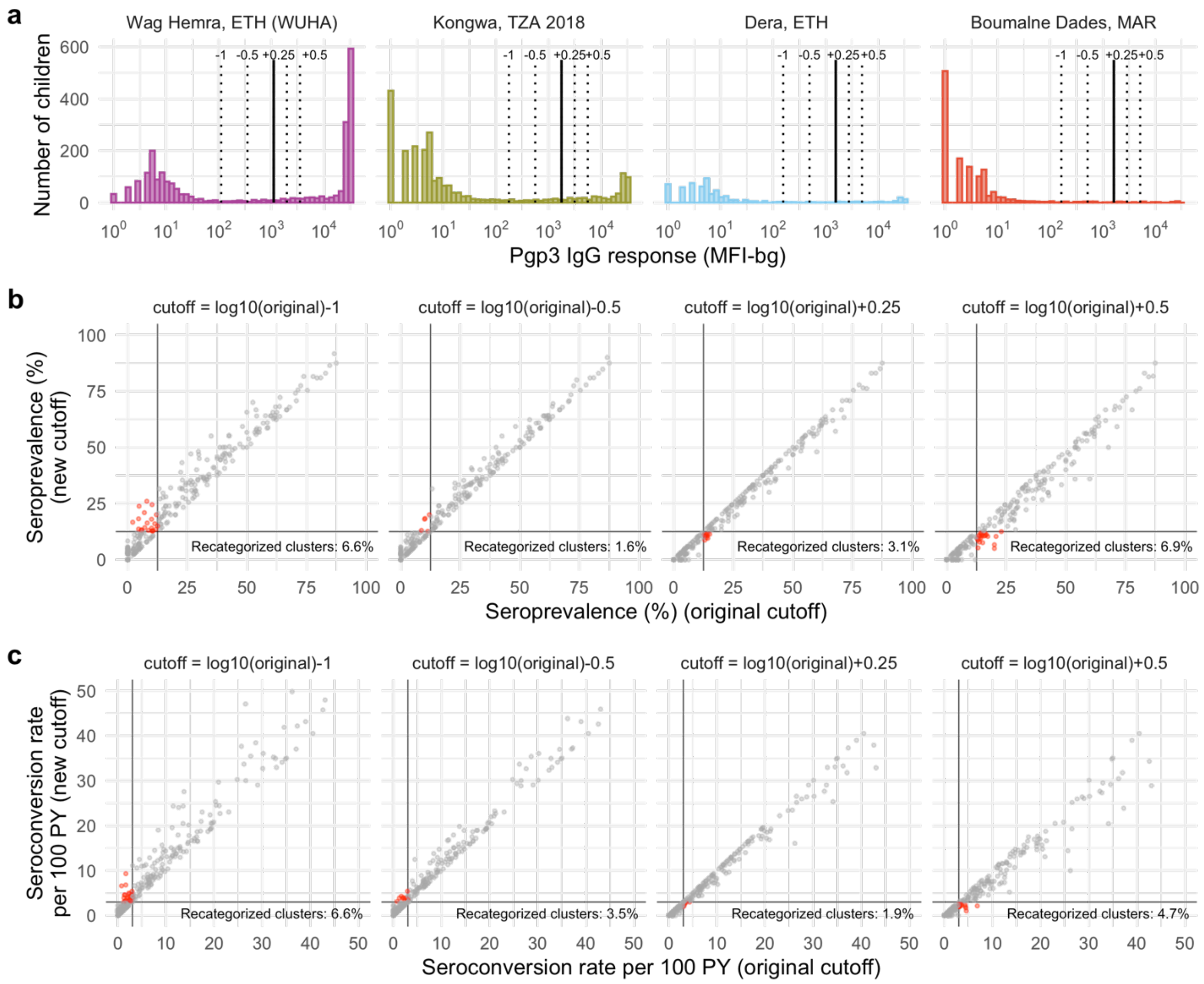

Supplementary Fig. 7. Robustness of seroprevalence and seroconversion rate to changes in seropositivity threshold. (**a**) Seroprevalence and seroconversion rate were recalculated using arbitrary seropositivity cutoffs at -1, -0.5, +0.25, and +0.5 in $\log_{10}$ units from the original cutoff, as shown in four populations representing a gradient of transmission settings. (**b)** At each new cutoff, we re-estimated seroprevalence and the proportion of clusters that were recategorized (red points) based on a threshold representing 90% sensitivity to identify clusters with any PCR infections (solid black lines, 12.5% seroprevalence, see **Fig 6**). (**c**) At each new cutoff, we re-estimated the seroconversion rate assuming no seroreversion and the proportion of clusters that were recategorized (red points) based on a threshold representing 90% sensitivity to identify clusters with any PCR infections (solid black lines, 2.75 seroconversions per 100 PY, see **Supplementary Fig 5**).

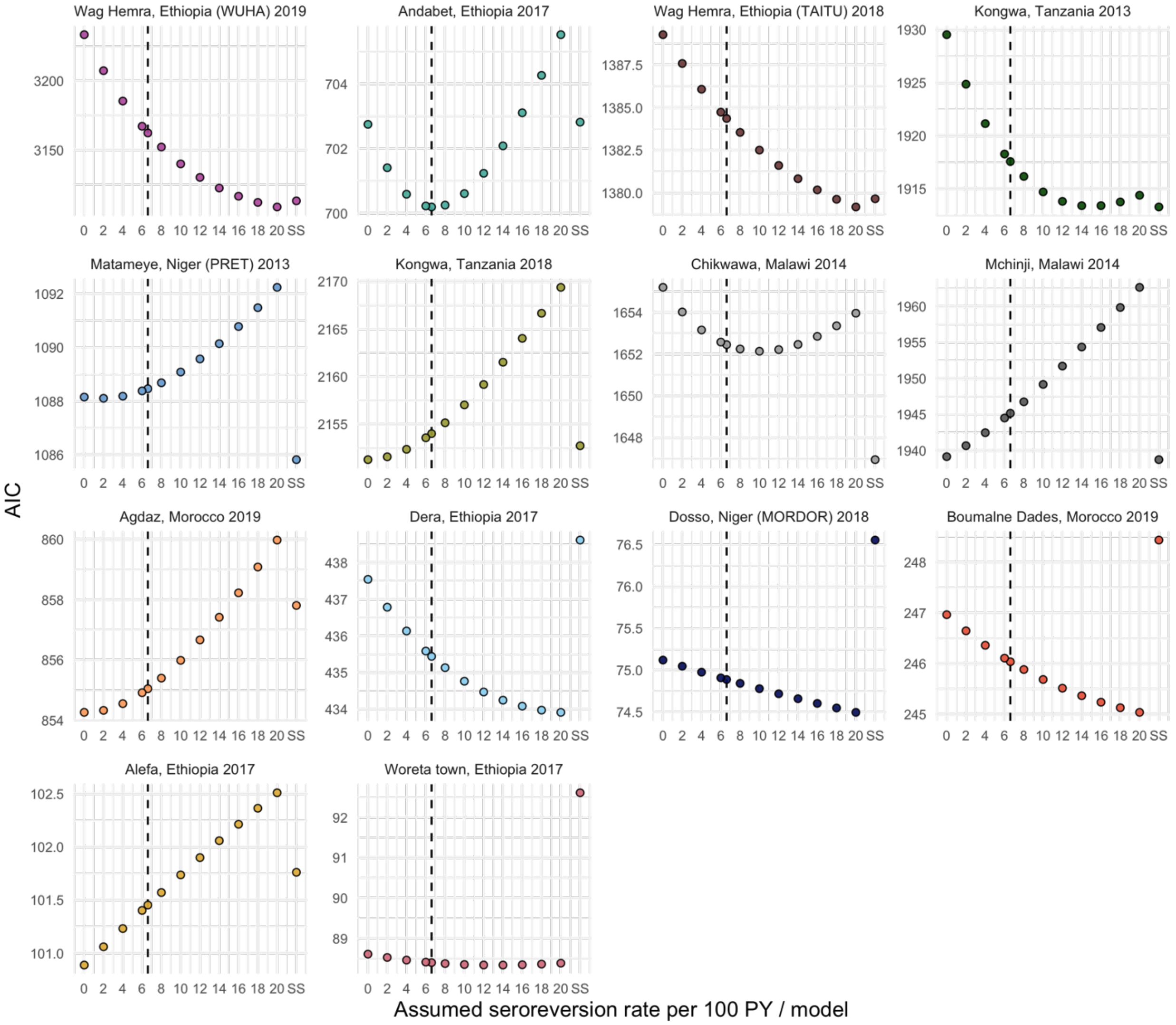

Supplementary Fig. 8. Seroconversion model comparison based on AIC for catalytic models with and without seroreversion and semiparametric spline model. In the main analysis, the catalytic model allowing for seroreversion assumed a seroreversion rate of 6.6 per 100 person-years and is marked with a vertical dashed line in each panel. The semiparametric spline model (SS) allows seroconversion rate to vary flexibly with age. Points may be missing in cases where models did not converge. Panels are arranged in order of descending population-level seroprevalence.

Supplementary Table 1. Overview of included studies.

Country	Study population	Admin-istrative level	Number of clusters[1]	Number of children	Study type	Year [2]	Trachoma program stage	Intervention context	Citation[3]
Ethiopia	Alefa	District	22	712	Survey	2017	From endemic to near elimination	Andabet, Dera, and Woreta town received MDA approximately 8 months prior to survey; Alefa had not received MDA for about 2.5 years	26
	Andabet	District	21	569					
	Dera	District	22	628					
	Woreta town	District	11	261					
Ethiopia	Wag Hemra (WUHA)	Area consisting of 3 contiguous districts	40	2287	RCT	2019	Endemic	2-arm RCT compared immediate and delayed intensive water, sanitation and hygiene intervention; annual MDA conducted for 8 years prior, but suspended in both arms during RCT	49
Ethiopia	Wag Hemra (TAITU)	Area consisting of 3 contiguous districts	37	1114	RCT	2018	Endemic	3-arm RCT compared MDA, triannual MDA to children 0–5-years-old with Ct infection, or delayed treatment; annual MDA conducted for 8 years prior to trial	48
Malawi	Chikwawa	District consisting of 3 EUs	71	2630	Survey	2014	Near elimination	MDA conducted annually from 2011 to 2013	31
	Mchinji	District consisting of 3 EUs	70	3271					
Morocco	Agdaz	District	28	1212	Survey	2019	Post-validation	MDA stopped in 2005	32,56
	Boumalne Dades	District	29	1266					
Niger	Dosso (MORDOR)	2 districts within the Dosso Region	29	939	RCT	2018	Post-validation	RCT compared placebo to azithromycin distributed to children <5y in intervention arm to prevent mortality	36
Niger	Matameye (PRET)	District	22	970	RCT	2013	Endemic	3-year 2x2 factorial RCT compared annual vs. 2x/year treatment and standard vs. enhanced coverage; serology collected only at final follow-up visit for enhanced coverage clusters	4,25
Tanzania	Kongwa	District	8	1577	RCT	2013	Near elimination	Area was highly endemic and received many rounds of MDA prior to study; RCT compared a single round of MDA at baseline to control; MDA was expanded in later years of the study	29

Country	Study population	Admin-istrative level	Number of clusters[1]	Number of children	Study type	Year [2]	Trachoma program stage	Intervention context	Citation[3]
Tanzania	Kongwa	District	50	2391	Survey	2018	Post-validation	2 years post-MDA	30

1 Clusters with Pgp3 IgG measurements in at least 15 children available for this analysis
2 Years included in this analysis
3. Citation numbers refer to citations in the article's main text
Abbreviations: RCT - randomized controlled trial; MDA - mass drug administration. EU – Evaluation Unit

Supplementary Table 2. Sensitivity and specificity to identify clusters with *C. trachomatis* infection by seroprevalence threshold among 1-9 year olds.

Seroprevalence threshold (%)	Sensitivity	Specificity
0.0	1.000	0.101
0.5	1.000	0.101
1.0	1.000	0.101
1.5	1.000	0.101
2.0	1.000	0.107
2.5	1.000	0.178
3.0	1.000	0.207
3.5	1.000	0.213
4.0	1.000	0.237
4.5	1.000	0.266
5.0	0.991	0.325
5.5	0.982	0.355
6.0	0.982	0.402
6.5	0.982	0.432
7.0	0.973	0.473
7.5	0.964	0.491
8.0	0.955	0.527
8.5	0.946	0.533
9.0	0.938	0.550
9.5	0.938	0.568
10.0	0.938	0.621
10.5	0.938	0.669
11.0	0.938	0.675
11.5	0.929	0.692
12.0	0.911	0.710
12.5	0.911	0.716
13.0	0.902	0.722
13.5	0.902	0.751
14.0	0.875	0.757
14.5	0.875	0.769
15.0	0.857	0.781
15.5	0.848	0.787
16.0	0.848	0.817
16.5	0.848	0.828
17.0	0.848	0.828
17.5	0.839	0.846

Seroprevalence threshold (%)	Sensitivity	Specificity
18.0	0.821	0.846
18.5	0.821	0.852
19.0	0.813	0.882
19.5	0.813	0.882
20.0	0.813	0.888
20.5	0.813	0.893
21.0	0.813	0.917
21.5	0.813	0.923
22.0	0.804	0.923
22.5	0.804	0.929
23.0	0.786	0.935
23.5	0.768	0.935
24.0	0.759	0.935
24.5	0.732	0.935
25.0	0.732	0.935
25.5	0.732	0.935
26.0	0.723	0.935
26.5	0.714	0.935
27.0	0.696	0.935
27.5	0.688	0.935
28.0	0.679	0.941
28.5	0.661	0.947
29.0	0.661	0.959
29.5	0.634	0.959
30.0	0.616	0.964
30.5	0.616	0.964
31.0	0.607	0.970
31.5	0.607	0.970
32.0	0.580	0.970
32.5	0.571	0.970
33.0	0.563	0.970
33.5	0.527	0.976
34.0	0.527	0.976
34.5	0.509	0.976
35.0	0.500	0.976
35.5	0.500	0.976
36.0	0.491	0.976
36.5	0.491	0.982

Seroprevalence threshold (%)	Sensitivity	Specificity
37.0	0.491	0.982
37.5	0.464	0.982
38.0	0.455	0.982
38.5	0.455	0.982
39.0	0.438	0.988
39.5	0.438	0.988
40.0	0.429	0.988
40.5	0.429	0.988
41.0	0.393	0.988
41.5	0.393	0.988
42.0	0.393	0.988
42.5	0.393	0.988
43.0	0.375	0.988
43.5	0.375	0.988
44.0	0.366	0.994
44.5	0.366	0.994
45.0	0.357	0.994
45.5	0.357	0.994
46.0	0.357	0.994
46.5	0.357	0.994
47.0	0.357	0.994
47.5	0.348	0.994
48.0	0.348	0.994
48.5	0.339	0.994
49.0	0.339	0.994
49.5	0.339	0.994
50.0	0.321	0.994
50.5	0.321	0.994
51.0	0.313	0.994
51.5	0.313	0.994
52.0	0.304	0.994
52.5	0.295	0.994
53.0	0.295	0.994
53.5	0.286	0.994
54.0	0.277	0.994
54.5	0.259	1.000
55.0	0.250	1.000
55.5	0.232	1.000

Seroprevalence threshold (%)	Sensitivity	Specificity
56.0	0.232	1.000
56.5	0.232	1.000
57.0	0.223	1.000
57.5	0.223	1.000
58.0	0.214	1.000
58.5	0.214	1.000
59.0	0.196	1.000
59.5	0.196	1.000
60.0	0.196	1.000
60.5	0.196	1.000
61.0	0.188	1.000
61.5	0.179	1.000
62.0	0.161	1.000
62.5	0.143	1.000
63.0	0.143	1.000
63.5	0.143	1.000
64.0	0.116	1.000
64.5	0.116	1.000
65.0	0.116	1.000
65.5	0.116	1.000
66.0	0.116	1.000
66.5	0.116	1.000
67.0	0.107	1.000
67.5	0.107	1.000
68.0	0.098	1.000
68.5	0.098	1.000
69.0	0.098	1.000
69.5	0.098	1.000
70.0	0.098	1.000
70.5	0.098	1.000
71.0	0.089	1.000
71.5	0.080	1.000
72.0	0.080	1.000
72.5	0.080	1.000
73.0	0.071	1.000
73.5	0.071	1.000
74.0	0.063	1.000
74.5	0.063	1.000

Seroprevalence threshold (%)	Sensitivity	Specificity
75.0	0.063	1.000
75.5	0.054	1.000
76.0	0.054	1.000
76.5	0.054	1.000
77.0	0.054	1.000
77.5	0.054	1.000
78.0	0.054	1.000
78.5	0.054	1.000
79.0	0.054	1.000
79.5	0.045	1.000
80.0	0.045	1.000
80.5	0.045	1.000
81.0	0.036	1.000
81.5	0.036	1.000
82.0	0.036	1.000
82.5	0.036	1.000
83.0	0.027	1.000
83.5	0.027	1.000
84.0	0.027	1.000
84.5	0.027	1.000
85.0	0.018	1.000
85.5	0.018	1.000
86.0	0.018	1.000
86.5	0.018	1.000
87.0	0.009	1.000
87.5	0.000	1.000
88.0	0.000	1.000
88.5	0.000	1.000
89.0	0.000	1.000

Supplementary Table 3. Sensitivity and specificity to identify clusters with *C. trachomatis* infection by seroconversion rate threshold among 1-9 year olds.

Seroconversion rate per 100 PY threshold (no seroreversion)	Sensitivity	Specificity
0.00	1.000	0.101
0.25	1.000	0.101
0.50	1.000	0.130
0.75	1.000	0.231
1.00	0.991	0.272
1.25	0.982	0.337
1.50	0.973	0.438
1.75	0.955	0.485
2.00	0.946	0.550
2.25	0.938	0.586
2.50	0.929	0.663
2.75	0.911	0.692
3.00	0.893	0.728
3.25	0.884	0.751
3.50	0.875	0.775
3.75	0.866	0.793
4.00	0.866	0.805
4.25	0.866	0.840
4.50	0.857	0.852
4.75	0.857	0.864
5.00	0.830	0.870
5.25	0.830	0.888
5.50	0.821	0.911
5.75	0.813	0.917
6.00	0.786	0.923
6.25	0.768	0.929
6.50	0.741	0.935
6.75	0.723	0.941
7.00	0.696	0.941
7.25	0.679	0.941
7.50	0.661	0.941
7.75	0.643	0.953
8.00	0.643	0.953
8.25	0.625	0.959
8.50	0.616	0.964
8.75	0.616	0.964

Seroconversion rate per 100 PY threshold (no seroreversion)	Sensitivity	Specificity
9.00	0.598	0.964
9.25	0.580	0.964
9.50	0.563	0.964
9.75	0.563	0.970
10.00	0.545	0.976
10.25	0.545	0.976
10.50	0.536	0.976
10.75	0.536	0.976
11.00	0.527	0.976
11.25	0.509	0.976
11.50	0.473	0.976
11.75	0.473	0.976
12.00	0.473	0.976
12.25	0.455	0.976
12.50	0.455	0.976
12.75	0.446	0.976
13.00	0.429	0.982
13.25	0.420	0.982
13.50	0.420	0.982
13.75	0.402	0.988
14.00	0.393	0.988
14.25	0.393	0.988
14.50	0.384	0.988
14.75	0.357	0.988
15.00	0.357	0.994
15.25	0.357	0.994
15.50	0.348	0.994
15.75	0.348	0.994
16.00	0.339	0.994
16.25	0.339	0.994
16.50	0.321	0.994
16.75	0.313	0.994
17.00	0.313	1.000
17.25	0.313	1.000
17.50	0.295	1.000
17.75	0.295	1.000
18.00	0.295	1.000
18.25	0.295	1.000

Seroconversion rate per 100 PY threshold (no seroreversion)	Sensitivity	Specificity
18.50	0.295	1.000
18.75	0.286	1.000
19.00	0.286	1.000
19.25	0.259	1.000
19.50	0.241	1.000
19.75	0.232	1.000
20.00	0.223	1.000
20.25	0.223	1.000
20.50	0.205	1.000
20.75	0.205	1.000
21.00	0.196	1.000
21.25	0.188	1.000
21.50	0.188	1.000
21.75	0.188	1.000
22.00	0.188	1.000
22.25	0.188	1.000
22.50	0.188	1.000
22.75	0.188	1.000
23.00	0.188	1.000
23.25	0.179	1.000
23.50	0.179	1.000
23.75	0.179	1.000
24.00	0.179	1.000
24.25	0.179	1.000
24.50	0.179	1.000
24.75	0.179	1.000
25.00	0.170	1.000
25.25	0.170	1.000
25.50	0.170	1.000
25.75	0.161	1.000
26.00	0.161	1.000
26.25	0.161	1.000
26.50	0.161	1.000
26.75	0.143	1.000
27.00	0.143	1.000
27.25	0.143	1.000
27.50	0.143	1.000
27.75	0.134	1.000

Seroconversion rate per 100 PY threshold (no seroreversion)	Sensitivity	Specificity
28.00	0.134	1.000
28.25	0.134	1.000
28.50	0.134	1.000
28.75	0.125	1.000
29.00	0.125	1.000
29.25	0.116	1.000
29.50	0.116	1.000
29.75	0.116	1.000
30.00	0.116	1.000
30.25	0.107	1.000
30.50	0.107	1.000
30.75	0.107	1.000
31.00	0.107	1.000
31.25	0.107	1.000
31.50	0.107	1.000
31.75	0.107	1.000
32.00	0.107	1.000
32.25	0.107	1.000
32.50	0.107	1.000
32.75	0.098	1.000
33.00	0.089	1.000
33.25	0.089	1.000
33.50	0.089	1.000
33.75	0.089	1.000
34.00	0.089	1.000
34.25	0.080	1.000
34.50	0.080	1.000
34.75	0.071	1.000
35.00	0.063	1.000
35.25	0.054	1.000
35.50	0.054	1.000
35.75	0.054	1.000
36.00	0.054	1.000
36.25	0.045	1.000
36.50	0.045	1.000
36.75	0.045	1.000
37.00	0.045	1.000
37.25	0.036	1.000

Seroconversion rate per 100 PY threshold (no seroreversion)	Sensitivity	Specificity
37.50	0.036	1.000
37.75	0.036	1.000
38.00	0.036	1.000
38.25	0.036	1.000
38.50	0.036	1.000
38.75	0.036	1.000
39.00	0.027	1.000
39.25	0.027	1.000
39.50	0.027	1.000
39.75	0.027	1.000
40.00	0.027	1.000
40.25	0.027	1.000
40.50	0.027	1.000
40.75	0.027	1.000
41.00	0.027	1.000
41.25	0.027	1.000
41.50	0.027	1.000
41.75	0.027	1.000
42.00	0.027	1.000
42.25	0.027	1.000
42.50	0.027	1.000
42.75	0.018	1.000
43.00	0.018	1.000
43.25	0.009	1.000
43.50	0.009	1.000
43.75	0.009	1.000
44.00	0.009	1.000
44.25	0.009	1.000
44.50	0.009	1.000
44.75	0.009	1.000
45.00	0.009	1.000
45.25	0.009	1.000
45.50	0.009	1.000
45.75	0.009	1.000
46.00	0.009	1.000
46.25	0.009	1.000
46.50	0.009	1.000
46.75	0.009	1.000

Seroconversion rate per 100 PY threshold (no seroreversion)	Sensitivity	Specificity
47.00	0.009	1.000
47.25	0.009	1.000
47.50	0.009	1.000
47.75	0.009	1.000
48.00	0.009	1.000
48.25	0.009	1.000
48.50	0.009	1.000
48.75	0.009	1.000
49.00	0.009	1.000
49.25	0.009	1.000
49.50	0.009	1.000
49.75	0.009	1.000
50.00	0.009	1.000

Supplementary Table 4. Sensitivity and specificity to identify clusters with *C. trachomatis* infection by seroprevalence threshold among 1-5 year olds.

Seroprevalence threshold (%)	Sensitivity	Specificity
0.0	1.000	0.102
0.5	1.000	0.102
1.0	1.000	0.102
1.5	1.000	0.102
2.0	1.000	0.108
2.5	1.000	0.180
3.0	1.000	0.210
3.5	1.000	0.216
4.0	1.000	0.240
4.5	1.000	0.269
5.0	0.991	0.329
5.5	0.981	0.359
6.0	0.981	0.407
6.5	0.981	0.437
7.0	0.972	0.473
7.5	0.963	0.491
8.0	0.953	0.527
8.5	0.944	0.533
9.0	0.935	0.551
9.5	0.935	0.569
10.0	0.935	0.623
10.5	0.935	0.671
11.0	0.935	0.677
11.5	0.925	0.689
12.0	0.907	0.707
12.5	0.907	0.713
13.0	0.897	0.719
13.5	0.897	0.749
14.0	0.869	0.754
14.5	0.869	0.766
15.0	0.850	0.778
15.5	0.841	0.784
16.0	0.841	0.814
16.5	0.841	0.826
17.0	0.841	0.826
17.5	0.832	0.844

18.0	0.813	0.844
18.5	0.813	0.850
19.0	0.804	0.880
19.5	0.804	0.880
20.0	0.804	0.886
20.5	0.804	0.892
21.0	0.804	0.916
21.5	0.804	0.922
22.0	0.794	0.922
22.5	0.794	0.928
23.0	0.776	0.934
23.5	0.757	0.934
24.0	0.748	0.934
24.5	0.720	0.934
25.0	0.720	0.934
25.5	0.720	0.934
26.0	0.710	0.934
26.5	0.701	0.934
27.0	0.692	0.934
27.5	0.682	0.934
28.0	0.673	0.940
28.5	0.654	0.946
29.0	0.654	0.958
29.5	0.636	0.958
30.0	0.617	0.964
30.5	0.617	0.964
31.0	0.607	0.970
31.5	0.607	0.970
32.0	0.579	0.970
32.5	0.570	0.970
33.0	0.561	0.970
33.5	0.523	0.976
34.0	0.523	0.976
34.5	0.514	0.976
35.0	0.505	0.976
35.5	0.505	0.976
36.0	0.495	0.976
36.5	0.495	0.982
37.0	0.495	0.982

37.5	0.467	0.982
38.0	0.458	0.982
38.5	0.458	0.982
39.0	0.439	0.988
39.5	0.439	0.988
40.0	0.430	0.988
40.5	0.430	0.988
41.0	0.402	0.988
41.5	0.402	0.988
42.0	0.402	0.988
42.5	0.402	0.988
43.0	0.383	0.988
43.5	0.383	0.988
44.0	0.374	0.994
44.5	0.374	0.994
45.0	0.374	0.994
45.5	0.374	0.994
46.0	0.374	0.994
46.5	0.374	0.994
47.0	0.374	0.994
47.5	0.364	0.994
48.0	0.364	0.994
48.5	0.355	0.994
49.0	0.355	0.994
49.5	0.355	0.994
50.0	0.336	0.994
50.5	0.336	0.994
51.0	0.327	0.994
51.5	0.327	0.994
52.0	0.318	0.994
52.5	0.308	0.994
53.0	0.308	0.994
53.5	0.299	0.994
54.0	0.290	0.994
54.5	0.271	1.000
55.0	0.262	1.000
55.5	0.243	1.000
56.0	0.243	1.000
56.5	0.243	1.000

57.0	0.234	1.000
57.5	0.234	1.000
58.0	0.224	1.000
58.5	0.224	1.000
59.0	0.206	1.000
59.5	0.206	1.000
60.0	0.206	1.000
60.5	0.206	1.000
61.0	0.196	1.000
61.5	0.187	1.000
62.0	0.168	1.000
62.5	0.150	1.000
63.0	0.150	1.000
63.5	0.150	1.000
64.0	0.121	1.000
64.5	0.121	1.000
65.0	0.121	1.000
65.5	0.121	1.000
66.0	0.121	1.000
66.5	0.121	1.000
67.0	0.112	1.000
67.5	0.112	1.000
68.0	0.103	1.000
68.5	0.103	1.000
69.0	0.103	1.000
69.5	0.103	1.000
70.0	0.103	1.000
70.5	0.103	1.000
71.0	0.093	1.000
71.5	0.084	1.000
72.0	0.084	1.000
72.5	0.084	1.000
73.0	0.075	1.000
73.5	0.075	1.000
74.0	0.065	1.000
74.5	0.065	1.000
75.0	0.065	1.000
75.5	0.056	1.000
76.0	0.056	1.000

76.5	0.056	1.000
77.0	0.056	1.000
77.5	0.056	1.000
78.0	0.056	1.000
78.5	0.056	1.000
79.0	0.056	1.000
79.5	0.047	1.000
80.0	0.047	1.000
80.5	0.047	1.000
81.0	0.037	1.000
81.5	0.037	1.000
82.0	0.037	1.000
82.5	0.037	1.000
83.0	0.028	1.000
83.5	0.028	1.000
84.0	0.028	1.000
84.5	0.028	1.000
85.0	0.019	1.000
85.5	0.019	1.000
86.0	0.019	1.000
86.5	0.019	1.000
87.0	0.009	1.000
87.5	0.000	1.000

Supplementary Table 5. Sensitivity and specificity to identify clusters with *C. trachomatis* infection by seroconversion rate threshold among 1-5 year olds.

Seroconversion rate per 100 PY threshold (no seroreversion)	Sensitivity	Specificity
0.00	1.000	0.102
0.25	0.981	0.281
0.50	0.981	0.281
0.75	0.981	0.287
1.00	0.981	0.329
1.25	0.972	0.395
1.50	0.953	0.479
1.75	0.935	0.557
2.00	0.935	0.593
2.25	0.925	0.617
2.50	0.916	0.659
2.75	0.907	0.689
3.00	0.888	0.743
3.25	0.888	0.772
3.50	0.879	0.790
3.75	0.879	0.802
4.00	0.879	0.832
4.25	0.869	0.844
4.50	0.860	0.856
4.75	0.841	0.856
5.00	0.813	0.880
5.25	0.813	0.898
5.50	0.813	0.910
5.75	0.813	0.910
6.00	0.794	0.916
6.25	0.794	0.922
6.50	0.785	0.928
6.75	0.776	0.928
7.00	0.757	0.934
7.25	0.757	0.934
7.50	0.738	0.934
7.75	0.729	0.940
8.00	0.720	0.940
8.25	0.701	0.946
8.50	0.692	0.952
8.75	0.682	0.952

9.00	0.664	0.952
9.25	0.654	0.952
9.50	0.645	0.958
9.75	0.626	0.964
10.00	0.617	0.964
10.25	0.617	0.970
10.50	0.607	0.970
10.75	0.607	0.970
11.00	0.598	0.970
11.25	0.598	0.970
11.50	0.561	0.970
11.75	0.533	0.970
12.00	0.533	0.970
12.25	0.523	0.970
12.50	0.523	0.970
12.75	0.505	0.970
13.00	0.495	0.970
13.25	0.486	0.970
13.50	0.486	0.970
13.75	0.477	0.976
14.00	0.477	0.976
14.25	0.477	0.976
14.50	0.458	0.976
14.75	0.449	0.976
15.00	0.439	0.982
15.25	0.439	0.982
15.50	0.430	0.982
15.75	0.430	0.988
16.00	0.421	0.988
16.25	0.421	0.988
16.50	0.411	0.988
16.75	0.411	0.994
17.00	0.411	0.994
17.25	0.411	0.994
17.50	0.402	0.994
17.75	0.393	0.994
18.00	0.393	0.994
18.25	0.383	0.994
18.50	0.383	0.994

18.75	0.374	0.994
19.00	0.364	0.994
19.25	0.346	0.994
19.50	0.327	0.994
19.75	0.318	0.994
20.00	0.318	0.994
20.25	0.308	0.994
20.50	0.290	1.000
20.75	0.271	1.000
21.00	0.262	1.000
21.25	0.262	1.000
21.50	0.262	1.000
21.75	0.262	1.000
22.00	0.262	1.000
22.25	0.262	1.000
22.50	0.262	1.000
22.75	0.262	1.000
23.00	0.252	1.000
23.25	0.243	1.000
23.50	0.243	1.000
23.75	0.243	1.000
24.00	0.234	1.000
24.25	0.234	1.000
24.50	0.224	1.000
24.75	0.224	1.000
25.00	0.215	1.000
25.25	0.215	1.000
25.50	0.215	1.000
25.75	0.206	1.000
26.00	0.206	1.000
26.25	0.206	1.000
26.50	0.206	1.000
26.75	0.178	1.000
27.00	0.178	1.000
27.25	0.178	1.000
27.50	0.178	1.000
27.75	0.168	1.000
28.00	0.168	1.000
28.25	0.168	1.000

28.50	0.168	1.000
28.75	0.168	1.000
29.00	0.168	1.000
29.25	0.159	1.000
29.50	0.159	1.000
29.75	0.159	1.000
30.00	0.159	1.000
30.25	0.150	1.000
30.50	0.140	1.000
30.75	0.140	1.000
31.00	0.140	1.000
31.25	0.140	1.000
31.50	0.131	1.000
31.75	0.131	1.000
32.00	0.131	1.000
32.25	0.131	1.000
32.50	0.131	1.000
32.75	0.131	1.000
33.00	0.131	1.000
33.25	0.131	1.000
33.50	0.131	1.000
33.75	0.131	1.000
34.00	0.131	1.000
34.25	0.121	1.000
34.50	0.121	1.000
34.75	0.112	1.000
35.00	0.103	1.000
35.25	0.093	1.000
35.50	0.093	1.000
35.75	0.093	1.000
36.00	0.093	1.000
36.25	0.093	1.000
36.50	0.093	1.000
36.75	0.093	1.000
37.00	0.093	1.000
37.25	0.084	1.000
37.50	0.084	1.000
37.75	0.084	1.000
38.00	0.084	1.000

38.25	0.084	1.000
38.50	0.084	1.000
38.75	0.084	1.000
39.00	0.075	1.000
39.25	0.075	1.000
39.50	0.075	1.000
39.75	0.065	1.000
40.00	0.065	1.000
40.25	0.065	1.000
40.50	0.065	1.000
40.75	0.065	1.000
41.00	0.065	1.000
41.25	0.065	1.000
41.50	0.065	1.000
41.75	0.065	1.000
42.00	0.065	1.000
42.25	0.065	1.000
42.50	0.065	1.000
42.75	0.065	1.000
43.00	0.065	1.000
43.25	0.056	1.000
43.50	0.056	1.000
43.75	0.056	1.000
44.00	0.047	1.000
44.25	0.047	1.000
44.50	0.047	1.000
44.75	0.047	1.000
45.00	0.047	1.000
45.25	0.047	1.000
45.50	0.047	1.000
45.75	0.047	1.000
46.00	0.047	1.000
46.25	0.037	1.000
46.50	0.037	1.000
46.75	0.037	1.000
47.00	0.037	1.000
47.25	0.037	1.000
47.50	0.037	1.000
47.75	0.037	1.000

48.00	0.037	1.000
48.25	0.037	1.000
48.50	0.037	1.000
48.75	0.019	1.000
49.00	0.019	1.000
49.25	0.019	1.000
49.50	0.019	1.000
49.75	0.019	1.000
50.00	0.019	1.000
